# Vimentin intermediate filaments control actin stress fiber assembly through GEF-H1 and RhoA

**DOI:** 10.1242/jcs.196881

**Published:** 2017-03-01

**Authors:** Yaming Jiu, Johan Peränen, Niccole Schaible, Fang Cheng, John E. Eriksson, Ramaswamy Krishnan, Pekka Lappalainen

**Affiliations:** 1 Institute of Biotechnology, P.O. Box 56, University of Helsinki, Helsinki 00014, Finland; 2 Faculty of Medicine, P.O. Box 63, University of Helsinki, Helsinki 00014, Finland; 3 Department of Emergency Medicine, Beth Israel Deaconess Medical Center, Harvard Medical School, Boston, MA 02215, USA; 4 Cell Biology, Biosciences, Faculty of Science and Engineering, Åbo Akademi University, FI-20520 Turku, Finland; 5 Turku Centre for Biotechnology, University of Turku and Åbo Akademi University, POB 123, FI-20521 Turku, Finland

**Keywords:** Vimentin, Intermediate filament, Actin, Stress fiber, RhoA, GEF-H1

## Abstract

The actin and intermediate filament cytoskeletons contribute to numerous cellular processes, including morphogenesis, cytokinesis and migration. These two cytoskeletal systems associate with each other, but the underlying mechanisms of this interaction are incompletely understood. Here, we show that inactivation of vimentin leads to increased actin stress fiber assembly and contractility, and consequent elevation of myosin light chain phosphorylation and stabilization of tropomyosin-4.2 (see Geeves et al., 2015). The vimentin-knockout phenotypes can be rescued by re-expression of wild-type vimentin, but not by the non-filamentous ‘unit length form’ vimentin, demonstrating that intact vimentin intermediate filaments are required to facilitate the effects on the actin cytoskeleton. Finally, we provide evidence that the effects of vimentin on stress fibers are mediated by activation of RhoA through its guanine nucleotide exchange factor GEF-H1 (also known as ARHGEF2). Vimentin depletion induces phosphorylation of the microtubule-associated GEF-H1 on Ser886, and thereby promotes RhoA activity and actin stress fiber assembly. Taken together, these data reveal a new mechanism by which intermediate filaments regulate contractile actomyosin bundles, and may explain why elevated vimentin expression levels correlate with increased migration and invasion of cancer cells.

## Abstract (precis)

**Summary:** Vimentin intermediate filaments control the activity of RhoA, and consequent stress fiber assembly and contractility by downregulating its guanine nucleotide exchange factor GEF-H1.

## INTRODUCTION

The actin cytoskeleton contributes to diverse cell biological, developmental, physiological and pathological processes in multicellular animals. Precisely regulated polymerization of actin filaments provides a force for generating membrane protrusions and invaginations during cell morphogenesis, migration and endocytosis. Actin and myosin II filaments also form contractile structures, where the force is generated by movement of myosin motor domains along actin filaments. The most prominent contractile actomyosin structures in non-muscle cells are stress fibers. Beyond cell migration and morphogenesis, stress fibers contribute to adhesion, mechanotransduction, endothelial barrier integrity and myofibril assembly (Burridge and Wittchen, 2013; Sanger et al., 2005; Tojkander et al., 2015; Wong et al., 1983; Yi et al., 2012). Stress fibers can be classified into three categories, which differ in their protein compositions and assembly mechanisms. Dorsal stress fibers are non-contractile actin bundles that are assembled through VASP- and formin-catalyzed actin filament polymerization at focal adhesions. Transverse arcs are contractile actomyosin bundles that are generated from the Arp2/3- and formin-nucleated lamellipodial actin filament network. These two stress fiber types serve as precursors for ventral stress fibers, which are mechanosensitive actomyosin bundles that are linked to focal adhesions at their both ends (Hotulainen and Lappalainen, 2006; Tojkander et al., 2011, 2015; Burnette et al., 2011; Skau et al., 2015; Tee et al., 2015). In addition to actin and myosin II, stress fibers are composed of a large array of actin-regulating and signaling proteins, including the actin filament cross-linking protein α-actinin and the actin filament-decorating tropomyosin proteins (Tojkander et al., 2012).

The Rho family small GTPases are central regulators of actin dynamics and organization in eukaryotic cells. Amongst these, RhoA in particular has been linked to generation of contractile actomyosin stress fibers. RhoA drives the assembly of focal adhesion-bound actomyosin bundles by inhibiting proteins that promote actin filament disassembly, by activating proteins that catalyze actin filament assembly at focal adhesions and by stimulating myosin II contractility through activation of ROCK kinases that catalyze myosin light chain phosphorylation (Heasman and Ridley, 2008). RhoA can be activated by Rho-guanine nucleotide exchange factors (Rho-GEFs), including Ect2, GEF-H1 (also known as ARHGEF2), MyoGEF (also known as PLEKHG6) and LARG (also known as ARHGEF12), which stimulate the GDP-to-GTP exchange in the nucleotide-binding pocket of RhoA. From these, Ect2 has a well-established role in the formation of contractile actomyosin structures at mitotic exit (Matthews et al., 2012), whereas the microtubule-associated GEF-H1 contributes to cell migration, cytokinesis and vesicular traffic (Ren et al., 1998; Nalbant et al., 2009; Birkenfeld et al., 2007; Pathak et al., 2012).

In addition to mechanosensitive interplay with focal adhesions and the plasma membrane, stress fibers interact with other cytoskeletal elements; microtubules and intermediate filament (IFs) (Huber et al., 2015; Jiu et al., 2015). IFs are stable but resilient cytoskeletal structures that provide structural support for cells and serve as signaling platforms. Vimentin and keratins are the major IF proteins in mesenchymal and epithelial cells, respectively (Eriksson et al., 2009; Snider and Omary, 2014; Loschke et al., 2015). Vimentin can interact with actin filaments both directly through its C-terminal tail and indirectly through the plectin cytoskeletal cross-linking protein (Esue et al., 2006; Svitkina et al., 1996). Furthermore, IFs display robust interactions with microtubules in cells (Huber et al., 2015). Importantly, several studies demonstrated that disruption of the actin cytoskeleton affects subcellular localization of the IF network in cells (Hollenbeck et al., 1989; Dupin et al., 2011; Jiu et al., 2015). More precisely, transverse arcs and ventral stress fibers interact with vimentin IFs through plectin, and retrograde flow of these contractile actomyosin bundles transports vimentin filaments from the leading edge towards the perinuclear region of the cell (Jiu et al., 2015). IFs can reciprocally affect actin-dependent processes such as cell adhesion and migration, because vimentin depletion results in impaired cell migration and pronounced stress fiber-attached focal adhesions (Bhattacharya et al., 2009; Eckes et al., 1998, 2000; Mendez et al., 2010). Moreover, keratin-8–keratin-18 displays interplay with Solo (also known as ARHGEF40), a RhoA-GEF, to control force-induced RhoA activation and consequent stress fiber assembly (Fujiwara et al., 2016). Finally, depletion of the cytoskeletal cross-linker, plectin, leads to similar abnormalities in focal adhesions and actin-dependent processes compared to vimentin depletion (Abrahamsberg et al., 2005; Andra et al., 1998). The effects of IFs and plectin on focal adhesions and the actin cytoskeleton have been so far linked to integrin-driven activation of focal adhesion kinase (FAK, also known as PTK2) and its downstream signaling cascade (Gregor et al., 2014). However, the effects of IFs on the stress fiber network and the underlying mechanisms have remained obscure. In addition, the principles by which vimentin controls cell adhesion, migration and invasion are incompletely understood.

Here, we report a vimentin-dependent downregulation of the stress fiber network in osteosarcoma cells and in fibroblasts. We show that depletion of vimentin results in an increased activation and phosphorylation of GEF-H1. This leads to an increase in the levels of active RhoA and consequent stress fiber assembly. Thus, our work proposes a novel pathway by which vimentin IFs regulate actin dynamics in cells.

## RESULTS

### Vimentin filaments inhibit the assembly of contractile stress fibers

**Fig. 1.**
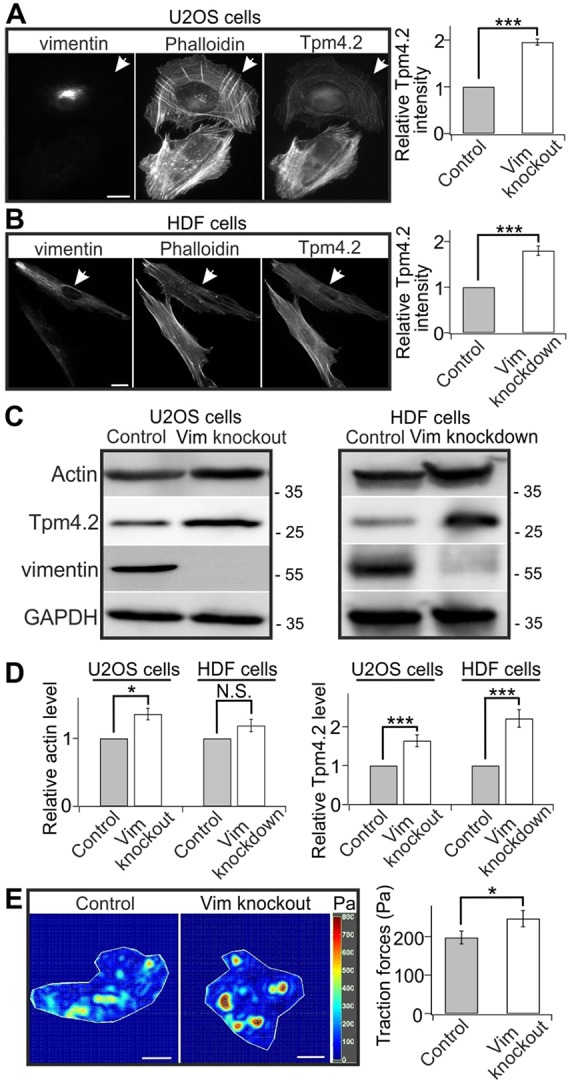
**Vimentin depletion induces stress fiber assembly.** (A,B) The intensities of Tpm4.2 and F-actin (as detected by fluorescent phalloidin) are increased in vimentin-knockout U2OS cells (A) and knockdown HDF cells generated using a vimentin siRNA pool (B). Panels on the left show representative images of control (arrowheads) and vimentin-depleted cells that were co-cultured on same plates. Panels on the right show the quantifications of normalized relative Tpm4.2 fluorescent intensities in control (A, 32 cells from nine images; B, 32 cells from nine images) and vimentin-knockout or knockdown cells (A, 38 cells from nine images; B, 33 cells from nine images). Mean intensity values of control and knockout or knockdown cells from each image were used for statistical analysis. ***P<0.001 (paired *t*-test). (C) Western blot analysis of actin and Tpm4.2 protein levels in control and vimentin-depleted U2OS (left panel) and HDF (right panel) cells. The blots were also probed with vimentin antibody to confirm that the vimentin-knockout U2OS cell culture is not contaminated by wild-type U2OS cells and to verify efficiency of vimentin depletion in siRNA-treated HDF cells, and with GADPH antibody to control equal sample loading. Molecular masses in kilodaltons (kDa) are indicated in the blots. (D) Quantification of the relative levels of actin (left panel) and Tpm4.2 (right panel) normalized to internal control GAPDH from five western blots. *P<0.05, ***P<0.001; N.S., not significant (paired *t*-test). (E) Vimentin-knockout results in increased cell contractility detected by traction force microscopy. Panels on the left show representative force maps of control and vimentin-knockout cells grown on 25 kPa polyacrylamide dishes with fluorescent nanobeads. The panel on the right shows the quantification of traction forces (root mean square traction) in control cells (*n*=47) and vimentin-knockout cells (*n*=47) from three independent experiments. *P<0.05 (Mann–Whitney–Wilcoxon rank-sum test). The data are presented as mean±s.e.m. Scale bars: 10 µm.

It is now well established that vimentin expression correlates with increased cell motility and invasiveness, which in turn are associated with actin dynamics. To this end, our recent work on U2OS cells revealed that actin transverse arcs transport vimentin filaments towards the cell center, whereas the vimentin IFs resist the retrograde movements of these contractile actomyosin bundles (Jiu et al., 2015). Interestingly, the vimentin-knockout U2OS cells also display thicker stress fibers as detected by phalloidin staining, and more intense tropomyosin 4.2 (for an explanation of tropomyosin nomenclature, see Geeves et al., 2015) staining compared to control cells (Fig. 1A). Tpm4.2 is a central stress fiber component that is involved in myosin II recruitment to stress fibers (Tojkander et al., 2011). To validate these findings, we performed western blot analysis on vimentin-knockout and control cells. Consistent with the immunofluorescence data, vimentin depletion resulted in a significant increase in Tpm4.2 protein levels, while the actin levels were only mildly increased upon vimentin depletion (Fig. 1C,D). The interplay between vimentin and Tpm4.2 is not restricted to U2OS cells, because immunofluorescence microscopy and western blot experiments also demonstrated increased Tpm4.2 levels in RNA interference (RNAi)-induced vimentin-knockdown human dermal fibroblasts (HDFs) (Fig. 1A–D).

Quantitative real-time RT-PCR (qRT-PCR) revealed no significant differences in Tpm4.2 mRNA levels between control and vimentin-knockout cells (Fig. S1A), indicating that vimentin does not regulate Tpm4.2 at the transcriptional level. We thus examined possible effects of vimentin on the stability of Tpm4.2 protein by treating U2OS cells with cycloheximide (CHX) to inhibit protein translation. This experiment showed that Tpm4.2 was very stable in CHX-treated vimentin-knockout cells during the 24 h experimental period, whereas in control cells Tpm4.2 protein levels were drastically diminished after a 6 h CHX-treatment (Fig. S1B). Moreover, global disruption of actin stress fiber network upon treatment with the myosin II inhibitor, blebbistatin, resulted in a significant decrease in the Tpm4.2 protein levels (Fig. S1C), indicating that Tpm4.2 protein is unstable and becomes degraded in the absence of stress fibers. Thus, lack of vimentin leads to an increased assembly and stability of stress fibers, and this consequently results in a diminished turnover of the Tpm4.2 protein.

**Fig. 2.**
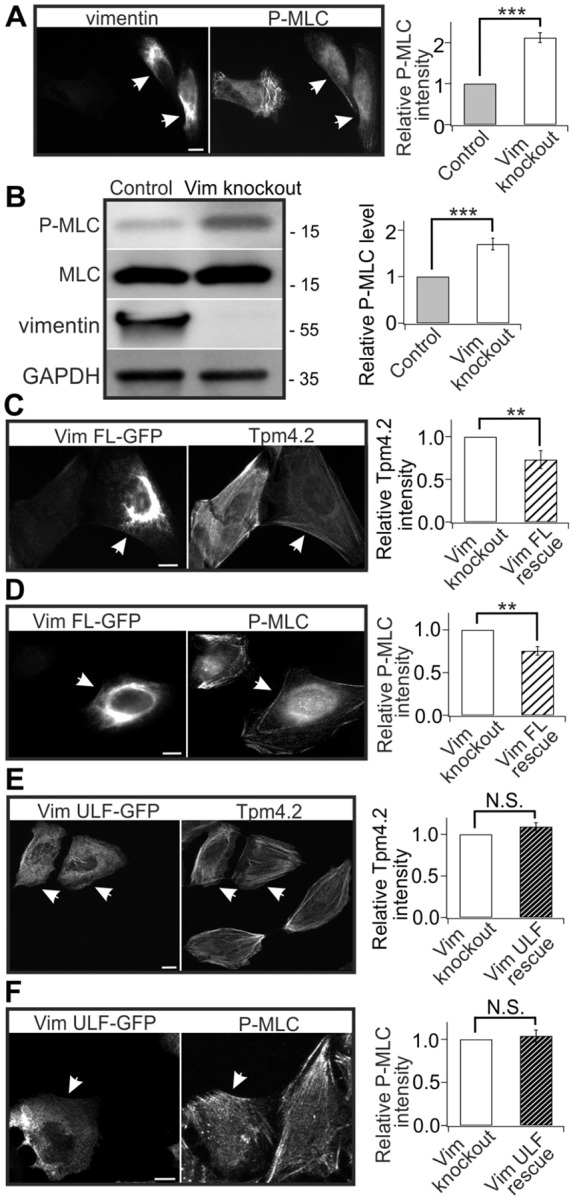
**The filamentous form of vimentin is necessary for its effects on stress fiber assembly.** (A) The intensity of P-MLC is increased in vimentin-knockout U2OS cells. The panel on the left shows representative images of control (arrowheads) and vimentin-knockout cells that were co-cultured on same plates. The panel on the right shows the quantification of normalized relative P-MLC fluorescent intensities in control (35 cells from nine images) and vimentin-knockout cells (37 cells from nine images). Mean intensity values of control and knockout cells from each image were used for statistical analysis. ***P<0.001 (paired *t*-test). (B) Western blot analysis of P-MLC levels in control and vimentin-knockout U2OS cells (left panel). The blots were also probed with vimentin antibody to confirm that the vimentin-knockout cell culture is not contaminated by wild-type cells, and with GADPH antibody to verify equal sample loading. Molecular masses in kilodaltons (kDa) are indicated in the blots. The panel on the right shows the normalized relative levels of P-MLC compared to the total MLC protein levels from three western blots. ***P<0.001 (paired *t*-test). (C,D) Full-length (FL) vimentin rescued the increase of Tpm4.2 (C) and P-MLC (D) levels induced by vimentin depletion. Panels on the left show representative images of vimentin-knockout cells expressing FL-vimentin–GFP (arrowheads) and non-transfected vimentin-knockout cells. Panels on the right show the quantifications of normalized relative Tpm4.2 (C, 33 control cells from eight images; 35 vimentin-knockout cells from eight images) and P-MLC (D, 26 control cells from eight images; 28 vimentin-knockout cells from eight images) fluorescence intensities. Mean intensity values of control and vimentin overexpression cells from each image were used for statistical analysis. **P<0.01 (paired *t*-test). (E,F) ‘Unit length form’ (ULF) vimentin is not able to rescue the increase of Tpm4.2 (E) and P-MLC (F) levels induced by vimentin depletion. Panels on the left show representative images of vimentin-knockout cells expressing ULF-vimentin–GFP (arrowheads) and non-transfected vimentin-knockout cells. Panels on the right show the quantifications of normalized relative Tpm4.2 (E, 27 control cells from eight images; 32 knockout cells from eight images) and P-MLC (F, 29 control cells from eight images; 28 knockout cells from eight images) fluorescent intensities. Mean intensity values of control and ULF vimentin overexpression cells from each image were used for statistical analysis. The data are presented as mean±s.e.m. N.S., not significant. Scale bars: 10 µm.

Because Tpm4.2 localizes specifically to myosin II-containing transverse arcs and ventral stress fibers (Tojkander et al., 2011), we hypothesized that loss of vimentin may also affect contractility of stress fibers. Contractile force measurements performed using traction force microscopy reveled that vimentin-knockout cells exert ∼25% greater traction forces compared to control cells (Fig. 1E). To examine the activity of myosin II, control and vimentin-knockout cells were stained with an antibody detecting phosphorylated (Thr18/Ser19) myosin light chain (P-MLC; recognizing phosphorylated myosin light chain 2). Lack of vimentin resulted in ∼2-fold increases in P-MLC intensity and total P-MLC levels when cells were grown on glass, or on a softer 33 kPa polyacrylamide substrates (Fig. 2A,B). Conversely, overexpression of vimentin reduced the intensities of Tpm4.2 and P-MLC (Fig. S2C,D).

To examine whether the filamentous form of vimentin is necessary for its effects on stress fibers, we performed knockout-rescue experiments with wild-type vimentin and with the ‘unit length filament’ (ULF) vimentin mutant Y117L, which preserves vimentin interaction with other components of the cytoskeleton, but cannot assemble into filaments (Meier et al., 2009). These experiments revealed that whereas full-length GFP–vimentin can rescue the stress fiber phenotype, the knockout cells expressing ‘non-polymerizable’ ULF–GFP displayed similar intensities of Tpm4.2 and P-MLC compared to non-transfected vimentin-knockout cells (Fig. 2C–F). Taken together, these data show that intact vimentin IFs diminish stress fiber assembly, contractility, myosin light chain phosphorylation and Tpm4.2 stability.

### Vimentin depletion results in increased levels of active RhoA

**Fig. 3.**
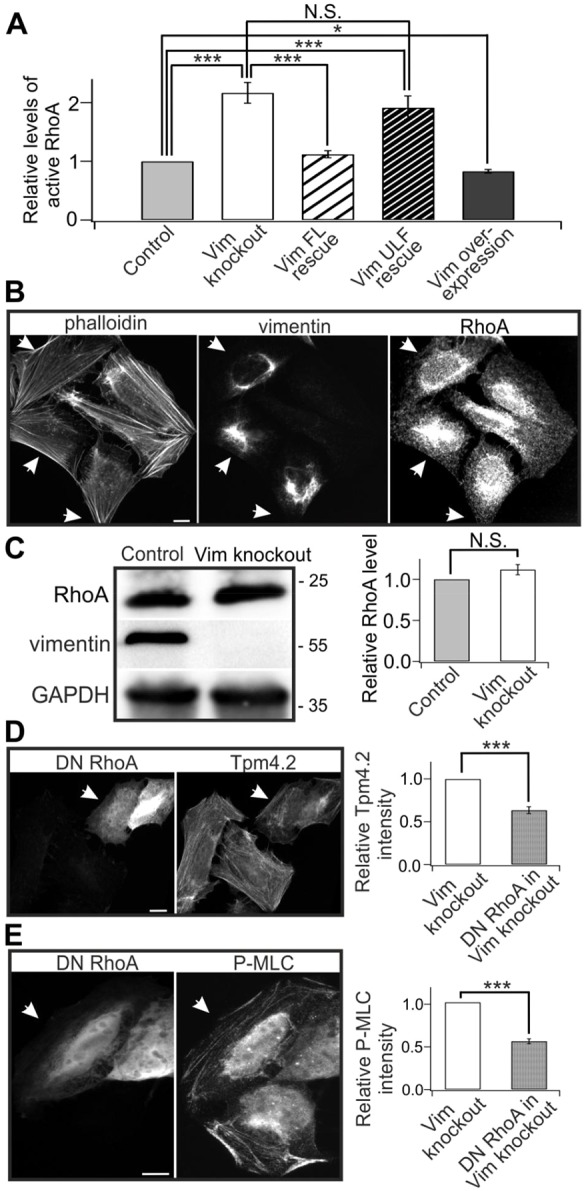
**Vimentin depletion increases the levels of active RhoA.** (A) G-LISA analysis of the levels of active RhoA in wild-type, vimentin-knockout, vimentin-knockout-rescue and vimentin overexpression U2OS cells. Data are from five independent experiments and were normalized to control cells. *P<0.05, ***P<0.001 (paired *t*-test). (B) Vimentin depletion does not drastically affect the subcellular localization of RhoA. Representative images show control (indicated by arrows) and vimentin-knockout cells co-cultured on same plates. (C) Western blot analysis of RhoA protein levels in control and vimentin-depleted U2OS cells. The blots were also probed with vimentin antibody to confirm that the vimentin-knockout cell culture is not contaminated by wild-type cells, and with GAPDH antibody to verify equal sample loading. Molecular masses in kilodaltons (kDa) are indicated in the blots. The panel on the right shows the quantified relative levels of RhoA protein normalized to internal control GAPDH from three western blots. (D,E) Expression of dominant negative (DN) RhoA inhibits the increase of Tpm4.2 (D) and P-MLC (E) levels in vimentin-knockout cells. Panels on the left show representative images of DN-RhoA-expressing cells (indicated by arrows) in a vimentin-knockout background. Panels on the right show the quantifications of normalized relative Tpm4.2 (D, 31 control cells from ten images; 25 DN-RhoA expressing cells from ten images) and P-MLC (E, 27 control cells from nine images; 29 DN-RhoA-expressing cells from nine images) fluorescence intensities. Mean intensity values of control and DN-RhoA overexpression cells from each image were used for statistical analysis. ***P<0.001 (paired *t*-test). The data are presented as mean±s.e.m. N.S., not significant. Scale bars: 10 µm.

RhoA regulates myosin light chain phosphorylation and activities of several actin-binding proteins to promote stress fiber contractility and assembly (Guilluy et al., 2011a,b; Lessey et al., 2012). We thus hypothesized that levels of active RhoA may be regulated by vimentin. By using G-LISA, a small GTPase activation assay, we discovered that absence of vimentin significantly increased the level of active GTP-bound, RhoA in U2OS cells (Fig. 3A). This effect could be rescued by expression of full-length vimentin, but not by the non-polymerizable ULF fragment, demonstrating that the presence of filamentous vimentin is required for suppression of RhoA activity (Fig. 3A). Based on immunostainings, western blot analysis and qRT-PCR, we observed that neither the subcellular localization nor the total protein levels and mRNA levels of RhoA were affected by vimentin depletion (Fig. 3B,C; Fig. S3D). This suggests that vimentin specifically controls the ratio of GTP- versus GDP-loaded RhoA. Vimentin also exerted its effects on stress fibers through RhoA activity; expression of dominant negative (DN)-RhoA blocked the augmentation of Tpm4.2 and P-MLC levels in both vimentin-knockout cells (Fig. 3D,E) and control U2OS cells (Fig. S3A–C). It is, however, important to note that DN-RhoA can compete for binding to Rho GDP dissociation inhibitors (GDIs) and may thus have different effects in cells compared to RhoA depletion. Taken together, these data demonstrate that vimentin filaments inhibit stress fiber assembly by downregulating RhoA.

### Vimentin regulates RhoA through GEF-H1

**Fig. 4.**
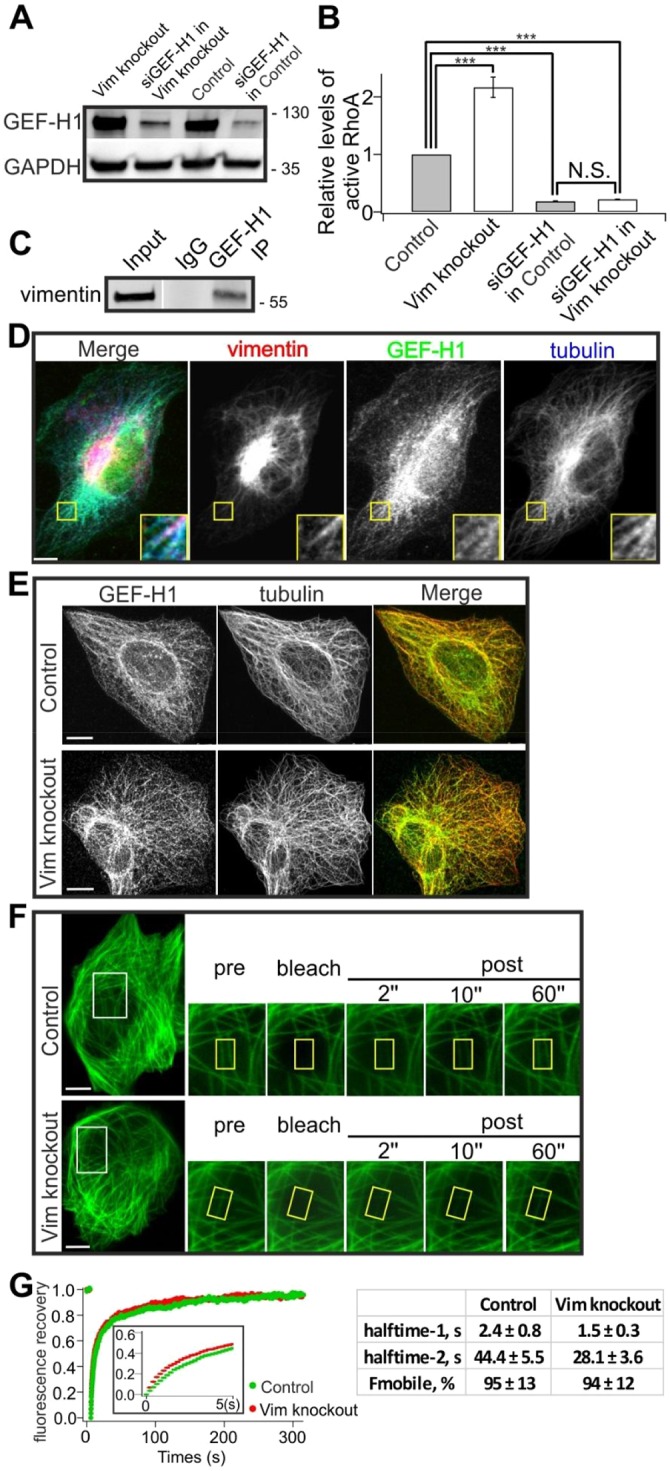
**GEF-H1 is critical for vimentin-mediated suppression of RhoA activity.** (A) Western blot demonstrating that GEF-H1 was efficiently silenced by siRNA (siGEF-H1) in both control and vimentin-knockout cells. The blot was also probed with GADPH antibody to verify equal sample loading. (B) G-LISA analysis of the levels of active RhoA in GEF-H1-silenced control and vimentin-knockout cells. The data are from five independent experiments and were normalized to results in control cells. ***P<0.001 (paired *t*-test). (C) Co-immunoprecipitation (IP) of GEF-H1 with vimentin from U2OS cell extracts. Whole-cell extracts were used for immunoprecipitation with an anti-GEF-H1 antibody, then probed with an anti-vimentin antibody. IgG is shown as a negative control. Molecular masses in kilodaltons (kDa) are indicated. (D) Image of a cell transfected with vimentin–mCherry, and stained with GEF-H1 and tubulin antibodies. Magnified regions from the area indicated by a yellow box demonstrate that vimentin filaments often colocalize with GEF-H1-containing microtubules. (E) Endogenous GEF-H1 displayed similar colocalization with microtubules in both control and vimentin-knockout cells. (F) Representative examples of GFP–GEF-H1 dynamics in control and vimentin-knockout cells as examined by FRAP. (G) Averaged recovery curves of the raw data are shown on the left (control *n*=15; vimentin knockout, *n*=17). The insert shows the recovery curves during the first 5 s following photobleaching. The averaged curves were fitted with double exponential equation, and mobile fractions and halftime values were calculated from the fitted data. The data are presented as mean±s.e.m. N.S., not significant. Scale bars: 10 µm.

Because IFs associate with microtubules (Leduc and Etienne-Manneville, 2015), we investigated by RNAi whether the microtubule-associated RhoA exchange factor GEF-H1 (Ren et al., 1998; Krendel et al., 2002) could mediate the cross-talk between vimentin and RhoA signaling during stress fiber formation and contractility. With appropriate siRNA oligonucleotide, we succeeded in efficiently depleting GEF-H1 from U2OS cells (Fig. 4A). Incubation of cells for 3 days with siRNAs against GEF-H1 led to changes in cell morphology and to a dramatic decrease in the number of stress fibers (data not shown), supporting the known role of GEF-H1 in stress fiber assembly. Strikingly, by using the G-LISA method, we found that silencing of GEF-H1 significantly diminished levels of active RhoA in both control and vimentin-knockout cells (Fig. 4B). This result was further confirmed with a different siRNA oligonucleotide against GEF-H1 (Fig. S3F,G). Thus, GEF-H1 appears to be the predominant GEF that activates RhoA in U2OS cells.

To elucidate the mechanism by which GEF-H1 is involved in vimentin-mediated suppression of RhoA, we assessed the interaction of endogenous vimentin and GEF-H1 by a co-immunoprecipitation assay. This experiment provided evidence that GEF-H1 either directly or indirectly interacts with vimentin (Fig. 4C). Furthermore, in cells transfected with vimentin–mCherry, and stained with anti-GEF-H1 and tubulin antibodies, vimentin filaments often aligned with GEF-H1-containing microtubules (Fig. 4D). We next examined whether vimentin regulates the localization or dynamics of GEF-H1 in cells. Immunofluorescence microscopy experiments demonstrated that GEF-H1 colocalized similarly with microtubules in both control and vimentin-knockout cells (Fig. 4E), whereas the dynamics of GEF-H1 were moderately increased in vimentin-knockout cells compared to control cells. Fluorescence recovery after photobleaching (FRAP) experiments on cells expressing GFP–GEF-H1 revealed a highly dynamic association of GEF-H1 with filamentous structures that are likely to represent microtubules. However, the recoveries of both the predominant dynamic population (halftime 1) and the smaller slow population (halftime 2) of GEF-H1 were more rapid in vimentin-knockout cells (halftimes 1 and 2 of ∼1.5 s and ∼28 s, respectively) compared to control cells (halftimes 1 and 2 of ∼2.4 s and ∼44 s, respectively), while the sizes of mobile fractions were very similar in both cases (95% and 94%, respectively) (Fig. 4F,G).

### Vimentin depletion results in increased activity and phosphorylation of GEF-H1

**Fig. 5.**
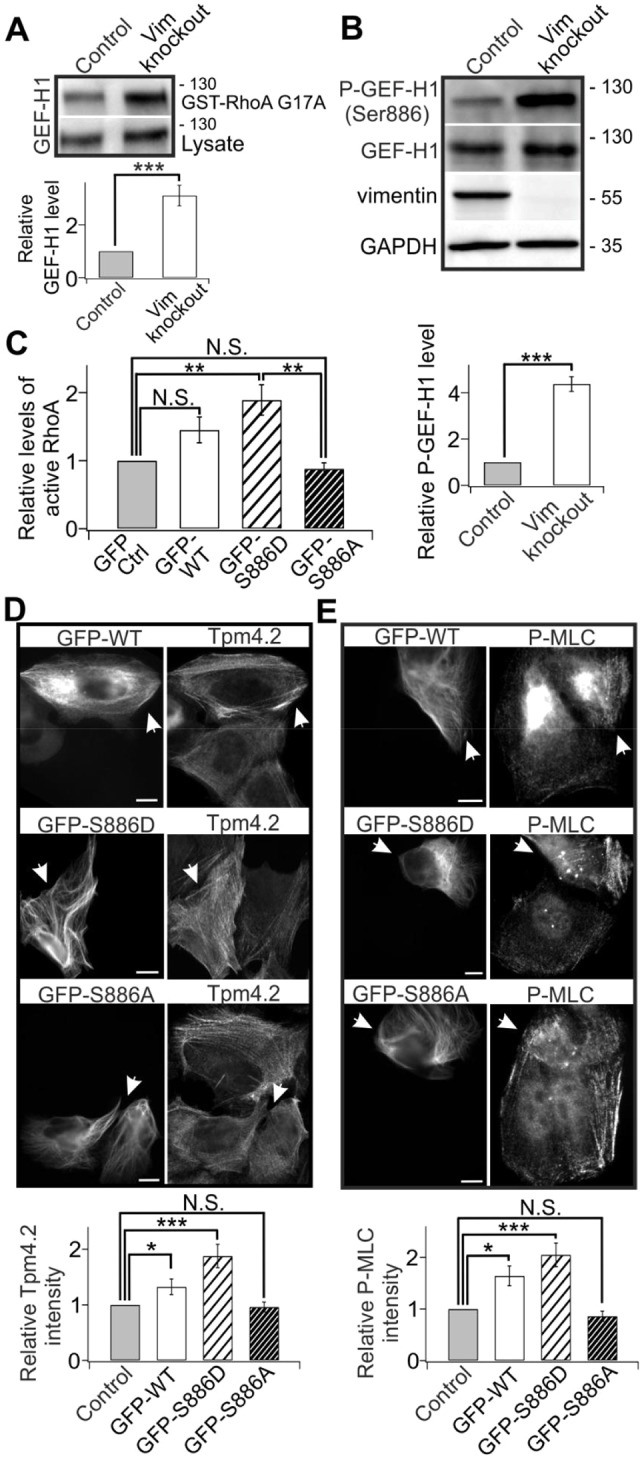
**Vimentin depletion results in increased activity and phosphorylation of GEF-H1.** (A) Active GEF-H1 was co-sedimented with GST–RhoA-G17A, and detected by western blotting using an anti-GEF-H1 antibody. The lower panel shows the quantification of normalized relative levels of GEF-H1 co-sedimenting with GST–RhoA-G17A compared to total GEF-H1 levels in cell lysates from five western blots. ***P<0.001 (paired *t*-test). (B) Western blot analysis of GEF-H1 phosphorylated on Ser886 and total GEF-H1 levels in control and vimentin-knockout cells. The blots were also probed with vimentin antibody to confirm that the vimentin-knockout cell culture is not contaminated by wild-type cells, and with GADPH antibody to verify equal sample loading. The lower panel shows the quantification of normalized relative levels of P-GEF-H1 (Ser886) compared to total GEF-H1 levels from five western blots. ***P<0.001 (paired *t*-test). Molecular masses in kilodaltons (kDa) are indicated. (C) G-LISA analysis of the levels of active RhoA in wild-type, phospho-mimic (S886D) and phospho-deficient (S886A) GEF-H1-expressing cells. The data are from five independent experiments and were normalized to control cells. **P<0.01 (paired *t*-test). (D) Tpm4.2 levels are increased in cells expressing the phospho-mimic (S886D) GEF-H1 mutant, but not in cells expressing the phospho-deficient (S886A) mutant. Upper panels show representative images of control cells and cells expressing wild-type or mutant GEF-H1 (arrowheads). The lower panel shows the quantification of normalized relative Tpm4.2 fluorescence intensities (wild-type GEF-H1: 32 control cells from nine images and 31 transfected cells from nine images; GEF-H1-S886D: 32 control cells from ten images and 35 transfected cells from total ten images; GEF-H1-S886A: 32 control cells from nine images and 38 transfected cells from nine images). Mean intensity values of control and GEF-H1 overexpression cells from each image were used for statistical analysis. *P<0.05; ***P<0.001 (paired *t*-test). (E) P-MLC levels are increased in cells expressing the phospho-mimic (S886D) GEF-H1 mutant, but not in cells expressing the phospho-deficient (S886A) mutant. Upper panels show representative images of control cells and cells expressing wild-type or mutant GEF-H1 (arrowheads). The lower panel shows the quantification of normalized relative P-MLC fluorescence intensities (wild-type GEF-H1: 35 control cells from nine images and 29 transfected cells from nine images; GEF-H1-S886D: 35 control cells from ten images and 33 transfected cells from ten images; GEF-H1-S886A: 35 control cells from nine images and 28 transfected cells from nine images). Mean intensity values of control and GEF-H1 overexpression cells from each image were used for statistical analysis. *P<0.05, ***P<0.001 (paired *t*-test). The data are presented as mean±s.e.m. N.S., not significant. Scale bars: 10 µm.

We next examined whether GEF-H1 activity is affected by vimentin. By using an activity assay that is based on co-sedimentation of GEFs with the GST–RhoA-G17A nucleotide-free mutant (Garcia-Mata et al., 2006), we revealed that the level of active GEF-H1 was ∼3**-**fold higher in vimentin-knockout cells compared to control cells (Fig. 5A). To elucidate the underlying mechanism, we examined the effects of vimentin depletion on phosphorylation of GEF-H1 on Ser886 (which is the only GEF-H1 phosphorylation site for which a commercial antibody is available). The specificity of the antibody was confirmed by western blot in cells expressing phosphorylation-deficient (S886A) GEF-H1 mutant (Fig. S4C). Strikingly, our results showed that GEF-H1 phosphorylation on Ser886 was strongly elevated in vimentin-knockout and knockdown versus control U2OS cells (Fig. 5B, Fig. S4B), whereas GEF-H1 protein and mRNA levels were not significantly altered (Fig. S3E). This mechanism is not specific to the cell type, because knockdown of vimentin from HDF cells resulted in a similar increase in GEF-H1 phosphorylation on Ser886 (Fig. S4A,B).

GEF-H1 phosphorylation on Ser886 was previously shown to induce 14-3-3 binding to the exchange factor, and relocation of 14-3-3 proteins to microtubules (Zenke et al., 2004). To determine whether GEF-H1 phosphorylation on Ser886 regulates the enzymatic activity of GEF-H1 in the context of actin stress fiber assembly, we examined the effects of wild-type GEF-H1 as well as the phosphomimetic (S886D) and phosphorylation-deficient (S886A) GEF-H1 mutants on RhoA activity in U2OS cells by using the G-LISA assay. Because transfection efficiency of these cells is quite high (>80%) for all GEF-H1 constructs, it was possible to examine the effects of these constructs on RhoA-activity using transiently transfected cells. Whereas wild-type GEF-H1-expressing cells displayed only a relatively small increase in the levels of active RhoA compared to control cells expressing GFP, overexpression of the phosphomimetic S886D mutant resulted in an almost 2-fold increase in the levels of active RhoA. The phosphorylation-deficient S886A mutant did not increase active RhoA over control levels (Fig. 5C). Furthermore, expression of the phosphomimetic S886D GEF-H1 resulted in increases in Tpm4.2 and P-MLC levels, whereas expression of the S886A GEF-H1 mutant had no detectable effects on Tpm4.2 or P-MLC levels (Fig. 5D,E). These data show that GEF-H1 phosphorylation on Ser886 increases its guanine nucleotide exchange activity towards RhoA, and consequently affects stress fiber assembly and contractility.

**Fig. 6.**
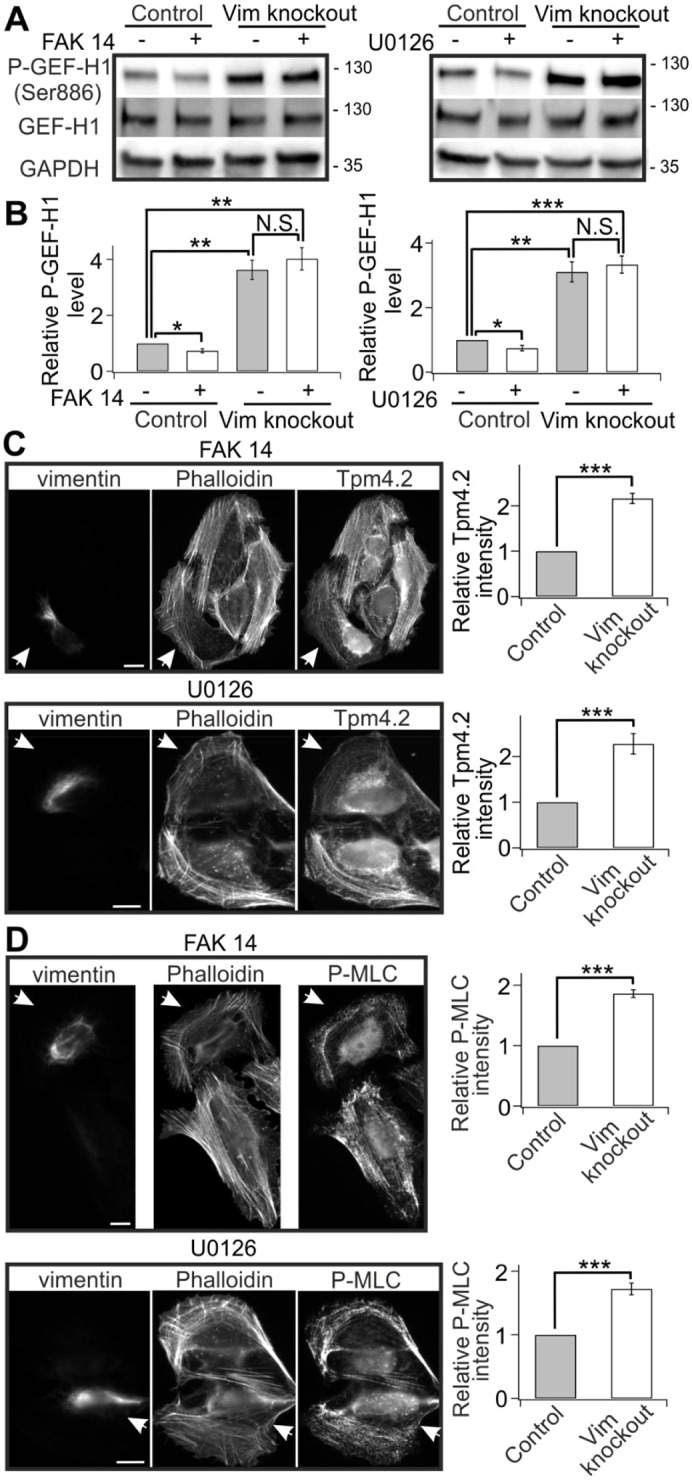
**GEF-H1 suppression by vimentin does not involve FAK and its downstream kinases.** (A) Western blot analysis of GEF-H1 phosphorylated on Ser886 and total GEF-H1 levels in control and vimentin-depleted U2OS cells incubated in the presence or absence of FAK inhibitor FAK-14 (left panel) or MEK inhibitor U0126 (right panel). The blots were also probed with GADPH antibodies to verify equal sample loading. Molecular masses in kilodaltons (kDa) are indicated. (B) Quantification of normalized relative levels of P-GEF-H1 (Ser886) compared to total GEF-H1 levels from five western blots for each condition. *P<0.05, **P<0.01, ***P<0.001 (paired *t*-test). (C,D) Immunostainings demonstrating that neither FAK-14 nor U0126 compounds could suppress the increased Tpm4.2 and P-MLC levels that were induced by depletion of vimentin. Wild-type cells in each panel are indicated with arrowheads. Panels on the right show the quantifications of normalized relative Tpm4.2 (C, FAK14: 28 control cells from six images and 29 vimentin-knockout cells from six images; U0126: 31 control cells from seven images and 28 vimentin-knockout cells from seven images) and P-MLC (D, FAK14: 33 control cells from seven images and 34 vimentin-knockout cells from seven images; U0126: 28 control cells from six images and 27 vimentin-knockout cells from six images) fluorescence intensities. Mean intensity values of control and vimentin-knockout cells from each image were used for statistical analysis. ***P<0.001 (paired *t*-test). The data are presented as mean±s.e.m. N.S., not significant. Scale bars: 10 µm.

Earlier studies provided evidence that IFs can affect stress fiber formation through plectin-mediated interactions with focal adhesions. The lack of vimentin or plectin were shown to lead to attenuation of the activities of FAK and its downstream kinases and upregulation of a compensatory feedback loop acting on RhoA (Gregor et al., 2014). FAK and its downstream kinases were, in other studies, demonstrated to regulate RhoA by activating its negative regulators RhoA GTPase-activating proteins (GAPs) (Aikawa et al., 2002; Schober et al., 2007). Based on western blot analysis, we found that neither the total protein levels nor the levels of active FAK and MEK1 and MEK2 (MEK1/2; also known MAP2K1 and MAP2K2, respectively) were drastically affected by vimentin depletion (Fig. S4D). To examine whether activation of FAK has effects on GEF-H1 phosphorylation on Ser886, or on vimentin-mediated attenuation of stress fiber assembly and contractility in U2OS cells, we used inhibitors of FAK (FAK-14) and its downstream kinases MEK1/2 (U0126), which diminished the levels of active phosphorylated kinases (Fig. S4E). While FAK-14 and U1026 slightly reduced the basal levels of GEF-H1 phosphorylation, neither of them inhibited the GEF-H1 phosphorylation on Ser886 induced by vimentin depletion (Fig. 6A,B). Furthermore, these compounds did not rescue the increased Tpm4.2 and P-MLC levels of vimentin-knockout cells (Fig. 6C,D). Taken together, we demonstrate that vimentin controls RhoA activity and stress fiber assembly through a novel GEF-H1-dependent pathway.

## DISCUSSION

Recent studies have demonstrated that the three cytoskeletal systems, actin filaments, microtubules and IFs, interact with each other, and exhibit interconnected functions in cell migration, morphogenesis and mechano-responsiveness (Huber et al., 2015). However, the mechanisms by which IFs affect the assembly and contractility of actin stress fibers have remained obscure. Here, we reveal that, first, vimentin filaments negatively regulate stress fiber assembly and contractility. Consequently, vimentin depletion results in accumulation of a central stress fiber component Tpm4.2 and increased myosin light chain phosphorylation. Second, vimentin filaments inhibit stress fiber assembly and contractility through down-regulating GEF-H1 and RhoA. Finally, vimentin controls GEF-H1 activity through modulating its phosphorylation on Ser886. Taken together, these data unravel new mechanisms by which vimentin IFs regulate the assembly and contractility of actomyosin bundles. Our results on the interplay between vimentin IFs and stress fibers may also explain why elevated expression levels of vimentin correlate with increased invasion and metastasis potential of cancer cells (e.g. Eckes et al., 1998; Mendez et al., 2010).

We show that vimentin depletion has comparable effects on stress fiber assembly, Tpm4.2 levels and GEF-H1 phosphorylation in both U2OS cells and dermal fibroblasts, indicating that the pathway by which vimentin regulates actin stress fibers is conserved in different mesenchymal cell types. However, whether the pathway identified here is also conserved in other animal cell types, including epithelial and endothelial cells, remains to be examined. Furthermore, our data provide evidence that Tpm4.2 is unstable in the absence of stress fibers, and thus the elevated Tpm4.2 levels in vimentin-depleted cells are due to increased assembly of stress fibers instead of more direct effects of vimentin, for example on Tpm4.2 transcription. Importantly, the vimentin–GEF-H1–RhoA–stress-fiber pathway identified here is different from the FAK-dependent compensatory feedback loop that was recently proposed to operate in the absence of vimentin and plectin (Gregor et al., 2014). This is because inhibitors against FAK and its downstream kinases could not rescue the effects of vimentin depletion on GEF-H1 phosphorylation, stress fiber assembly and contractility, although they resulted in a small decrease in GEF-H1 phosphorylation levels in control cells. Furthermore, earlier studies proposed that vimentin and plectin depletions regulate RhoA by attenuating the activity of FAK, which in turn leads to downregulation of both ARHGAP35 and ARHGAP5 (also known as p190RhoGAP) (Schober et al., 2007), rather than upregulation of GEF-H1 as shown by our results. Finally, plectin-null cells were shown to exert lower tractions to their environment compared to control cells (Schober et al., 2007; Gregor et al., 2014), whereas our study demonstrates that vimentin depletion increases contractility. Thus, inactivation of plectin and vimentin manifest differently on stress fibers and mechanical regulation of the cell. We do not rule out other mechanisms by which vimentin may control stress fiber assembly, for example, through regulating Rac1 activity via its GEF VAV2 at focal adhesions (Havel et al., 2015). Neither do we rule out other confounding factors including cell density and soft substrates that independently impact vimentin-dependent cell spreading and contractility (Mendez et al., 2014). In this regard, we emphasize that our findings were concordant between cells on glass and 33 kPa substrates.

How does vimentin control the GEF-H1 activity? First, we showed that GEF-H1 localization to microtubules is not drastically altered upon vimentin depletion. Next, by focusing on the GEF-H1 phosphorylation in control and vimentin-knockout cells, we showed that GEF-H1 phosphorylation on Ser886 was significantly increased in vimentin-deficient cells. Although previous studies demonstrated that GEF-H1 phosphorylation simultaneously on both Ser886 and Ser959 or only on Ser959 inhibit its activity (Birkenfeld et al., 2007; Yamahashi et al., 2011; Von Thun et al., 2013), our experiments using a phosphomimetic mutant protein provided evidence that GEF-H1 phosphorylation on Ser886 in U2OS cells results in its activation. Various kinases, including PAKs, Aurora A, Cdk1 and PAR1b (also known as MARK2) can inactivate GEF-H1 by phosphorylating inhibitory sites (Birkenfeld et al., 2007; Callow et al., 2005; Yamahashi et al., 2011), whereas extracellular signal-regulated kinase 1 and 2 (ERK1/2, also known as MAPK3 and MAPK1, respectively) phosphorylates GEF-H1 at the activating site Thr678 (Fujishiro et al., 2008; Guilluy et al., 2011a,b). Thus, as with many GEFs, regulation of GEF-H1 is a complex process, involving phosphorylations on several different sites that affect interactions of the protein with other kinases and interaction partners. These interaction partners may further activate GEF-H1 as demonstrated by a recent study in which the bacterial translocation type III secretion effector VopO was shown to bind GEF-H1 and consequently active the RhoA–ROCK pathway and actin stress fiber formation (Hiyoshi et al., 2015).

The mechanism by which vimentin downregulates phosphorylation of GEF-H1 on Ser886 can either depend on the availability of GEF-H1 for phosphorylation or from activation of a specific kinase in the absence of vimentin. Previous studies demonstrated that PAK1 phosphorylates GEF-H1 on Ser886 (Zenke et al., 2004). PAK1 silencing also attenuates vimentin phosphorylation on Ser56, and vimentin phosphorylation on Ser56 inversely regulates PAK1 activity in smooth muscle cells stimulated by 5-hydroxytryptamine (5-HT; serotonin) (Li et al., 2006). However, whether vimentin can regulate PAK1 and its downstream signaling in non-muscle cells has not been reported. Our western blot analysis indicated that neither PAK1 protein levels nor its activity (as detected by PAK1 phosphorylation on Thr423) were increased by vimentin depletion in U2OS cells. In addition, PAK1 inhibitor (IPA-3) did not affect the levels of Ser886 phosphorylated GEF-H1 in vimentin-knockout cells (Fig. S4F), indicating that PAK1 is not involved and that the signaling cascade in vimentin-mediated GEF-H1 regulation is more complex and is likely to involve other kinases. Moreover, absence of vimentin resulted in a small, but reproducible, increase in the GEF-H1 dynamics (Fig. 4F,G). Therefore, it is also possible that vimentin depletion does not activate specific kinases, but instead makes GEF-H1 more available for these kinases due to its increased dynamics.

Vimentin is a well-characterized biomarker of epithelial–mesenchymal transitions (EMTs). Several studies on multiple tumor types demonstrated that vimentin is specifically expressed in invasive cell lines, but not in stationary cancer cells (Singh et al., 2003; Hu et al., 2004; Wei et al., 2008). Furthermore, absence of vimentin was shown to lead to decreased cell migration speed and directionality (Eckes et al., 1998, 2000). Our work demonstrating effects of vimentin on GEF-H1 and RhoA activity, and downstream stress fiber assembly and contractility, may provide an explanation for these observations. Because extensive stress fibers enhance adhesion and inhibit cell motility, we speculate that upregulation of vimentin stimulates cell migration at least partially through inhibiting stress fiber assembly and contractility. In this context, it is important to note that altered GEF-H1 activity and expression levels have been linked to cancer progression (Cheng et al., 2012; Cullis et al., 2014; Biondini et al., 2015). Thus, in the future it will be interesting to examine how the vimentin–GEF-H1–RhoA pathway identified in our study contributes to the role of vimentin in cell migration and invasion *in vivo*.

## MATERIALS AND METHODS

### Cell culture and transfections

Human osteosarcoma (U2OS) cells and human dermal fibroblasts (HDFs) were maintained as described in Jiu et al. (2015). Vimentin-knockout U2OS cells were generated using CRISPR/Cas9 methods (Jiu et al., 2015). Transient transfections were performed with FuGENE HD transfection reagent (Promega) according to the manufacturer's instructions. Cells were subsequently incubated for 24 h and either fixed with 4% PFA (for GEF-H1 antibody staining, cells were fixed with methanol) or used for FRAP by detaching the cells with trypsin-EDTA and plating them on fibronectin-coated (10 μg/ml) glass-bottomed dishes (MatTek). For siRNA silencing, pre-annealed 3′ oligonucleotide duplexes were transfected into cells on 35 mm plates by using Lipofectamine RNAiMAX transfection reagent (Invitrogen) according to the manufacturer's instructions. Cells were incubated for 72 h for efficient depletion of the target proteins. For the cyclohexamide experiment, both wild-type and vimentin-knockout cells were treated with 20 μg/ml cyclohexamide (Sigma) in complete Dulbecco's modified Eagle's medium (DMEM) and harvested at corresponding time points. For the drug experiments, cells were treated with 10 µM blebbistatin (Sigma) for 30 min, 2 µM FAK inhibitor FAK14 (Tocris) for 30 min, 25 µM MEK inhibitor U0126 (Tocris) for 30 min, or 30 µM PAK1 inhibitor IPA-3 (Tocris) for 1 h before they were harvested for western blot analysis or fixed for immunofluorescence.

### Plasmids and siRNA oligonucleotides

Cloning strategy for constructs expressing GFP-tagged full-length vimentin or ‘unit length filament’ (ULF) and vimentin–mCherry are described in Yoon et al. (1998) and Eriksson et al. (2004). Dominant negative pRK5myc RhoA N19 (deposited by Alan Hall, Addgene plasmids #15900 and #15901). Wild-type GFP-GEF-H1, phosphomimetic S886D GFP-GEF-H1 and phosphorylation-deficient S886A GFP-GEF-H1 were kind gifts from Katalin Szaszi (St. Michael's Hospital, Toronto, Canada). pGEX-4T1-RhoA G17A was a gift from Rafael Garcia-Mata (Addgene plasmid # 69357). The ON-TARGET siRNA-SMART pool L-003551-00-0005 was used for vimentin knockdown in HDF cells and the siRNA target sequence 5′-UCACGAUGACCUUGAAUAA-3′ was used for vimentin knockdown in U2OS and HDF cells (Dharmacon). The siRNA target sequences 5′-GACUCAGACUCUAGCCAGA-3′ and 5′-CAGAUGUGUAAGACCUACU-3′ were used for GEF-H1 knockdown (Bioneer). AllStars Negative Control siRNA (Qiagen) was used as a control siRNA.

### Western blotting

Cells were washed three times with cold PBS, scraped, and lysed in Laemmli sample buffer (LSB) with 0.3 mM PMSF and protease and phosphatase inhibitor cocktail (Pierce). Protein concentrations were measured by using Bradford reagent (Sigma-Aldrich). 5% milk was used in blocking and washes were done by using TBST buffer (Tris-buffered saline, 0.1% Tween 20). Antibodies were used with the following dilutions in 5% BSA: vimentin rabbit polyclonal D21H3 antibody (dilution 1:1000; #5741, Cell Signaling); Tpm4.2 mouse monoclonal LC24 antibody (dilution 1:500; a kind gift from Peter W. Gunning, UNSW Australia); phospho-myosin light chain 2 (Thr18/Ser19) rabbit polyclonal antibody (dilution 1:500; #3674, Cell Signaling); myosin light chain mouse monoclonal antibody (dilution 1:1000; #M4401, Sigma); GEF-H1 rabbit monoclonal 55B6 antibody (dilution 1:1000; #4076, Cell Signaling); phospho-GEF-H1 (Ser886) rabbit monoclonal E1L6D antibody (dilution 1:1000; #14143, Cell Signaling); actin mouse polyclonal AC40 antibody (dilution 1:1000; #A4700, Sigma); RhoA rabbit polyclonal antibody (dilution 1:1000; #SAB2102002, Sigma); FAK rabbit polyclonal antibody (dilution 1:1000; #3285, Cell Signaling); phospho-FAK (Tyr397) rabbit polyclonal antibody (dilution 1:1000; #3283, Cell Signaling); MEK1/2 mouse polyclonal L38C12 antibody (dilution 1:1000; #4694, Cell Signaling); phospho-MEK1/2 (Ser217/221) rabbit polyclonal 41G9 antibody (dilution 1:1000; #9154, Cell Signaling); PAK1 rabbit polyclonal antibody (dilution 1:1000; #2602, Cell Signaling); phospho-PAK1 (Thr423) rabbit polyclonal antibody (dilution 1:1000; #2601, Cell Signaling); GAPDH mouse polyclonal antibody (dilution 1:1000; G8795, Sigma). Horseradish peroxidase (HRP)-linked secondary antibodies were used and chemiluminescence was measured after applying western blotting ECL spray (Advansta). The ImageJ program was applied to measure the band intensities of blots. In the quantifications, we calculated the intensity ratios of phosphorylated protein to protein of interest versus total protein to the internal control GAPDH. The values of control cells were set to 1 in each experiment, and the differences between the control and the knockout, knockdown or drug treatment cells in corresponding blots were calculated. The statistical differences between the two groups were assessed using the paired *t*-test. *P*<0.05 was considered significant.

### Immunofluorescence microscopy

Immunofluorescence experiments were performed as previously described (Jiu et al., 2015). Briefly, the cells were fixed with 4% PFA, washed three times with 0.2% BSA in Dulbecco's phosphate buffered saline and permeabilized with 0.1% Triton X-100 in PBS. The following primary antibodies were used: vimentin rabbit polyclonal D21H3 antibody (dilution 1:100; #5741, Cell Signaling) when cells were co-stained with Tpm4.2 antibody; vimentin mouse polyclonal V9 antibody (dilution 1:100; #V6630, Sigma) when cells were co-stained with P-MLC antibody; Tpm4.2 mouse monoclonal LC24 antibody (dilution 1:150; a kind gift from Peter W. Gunning, UNSW Australia); phospho-myosin light chain 2 (Thr18/Ser19) rabbit polyclonal antibody (dilution 1:100; #3674, Cell Signaling); RhoA rabbit polyclonal antibody (dilution 1:100; #SAB2102002, Sigma); GEF-H1 rabbit polyclonal antibody (dilution 1:50; #ab155785, Abcam); tubulin mouse monoclonal antibody (dilution 1:100; #4026, Sigma). Secondary antibodies were conjugated to Alexa Fluor 488, 568 or 647 (Invitrogen). F-actin was visualized with Alexa Fluor 488-, 568- or 647 conjugated to phalloidin (dilution 1:200; Invitrogen). Cells were imaged either with a wide-field fluorescence microscope (Leica DM6000) with a HCXPL APO 63×1.40-0.60 NA oil objective or by Leica TCS SP5 laser scanning confocal microscope with a 63×1.3 NA glycerol objective. Because the GEF-H1 antibody from Cell Signaling used for all western blot experiments does not work in immunofluorescence, we used GEF-H1 antibody from Abcam for immunofluorescence experiments where cells were fixed by methanol for 5 min at −20°C. For comparing Tpm4.2, P-MLC and RhoA levels, the control and vimentin-knockout or knockdown cells were mixed and distinguished from each other by vimentin antibody staining. Please note that vimentin, Tpm4.2, P-MLC, RhoA and GEF-H1 levels were constant between different control cells (data not shown). In the quantifications, we first measured the intensities (corresponding to expression levels) of a protein of interest in all control cells from individual immunofluorescence images and set the mean value to 1. Subsequently, the values from all knockout or knockdown cells from the same image were compared to the mean value obtained from the control cells. The mean intensity values of knockout or knockdown cells (normalized to the values obtained from control cells) from individual immunofluorescence images were used for the statistical analysis. s.e.m. represents the variation of mean intensities between individual immunofluorescence images. The statistical differences between two groups was assessed by using a paired *t*-test. *P*<0.05 was considered significant.

### FRAP

To analyze the kinetics of GEF-H1, wild-type and vimentin-knockout cells were transfected with GFP–GEF-H1 and incubated for 24 h. Confocal images were acquired with a 3I Marianas imaging system (3I Intelligent Imaging Innovations), consisting of an inverted spinning disk confocal microscope Zeiss Axio Observer Z1 (Zeiss), a Yokogawa CSU-X1 M1 confocal scanner and 63×1.2 NA WC-Apochromat Corr WD=0.28 M27 objective (Zeiss). A heated sample environment (+37°C) and CO_2_ control were used. SlideBook 6.0 software (3I Intelligent Imaging Innovations) was used for the image acquirement. Five pre-bleach images were acquired followed by bleaching scans with 100% intensity laser lines over the region of interest. Recovery of fluorescence was monitored 50 times every 200 ms and 300 times every 1 s. The intensity of the bleached area was normalized to a neighboring non-bleached area. Mean scatter plots were calculated from different FRAP experiments and the data were fitted with SigmaPlot 11.0 to f=a×(1×exp(×b×x))+c×(1−exp(−d×x)) double exponential equations. Recovery halftimes were obtained for each recovery curve and the means and standard deviations were calculated.

### Quantitative real-time RT-PCR

The total RNA was extracted from U2OS cells using RNeasy mini kits (Qiagen). cDNA was obtained by reverse-transcribing the same amount of total RNA using High Capacity cDNA Reverse Transcription Kit (Applied Biosystems). The complementary DNA products were amplified using sequence-specific primers for Tpm4.2 (forward, 5′-AGAAAGCGCTGAGGACAAG-3′; reverse, 5′-TTGGTGAGCCCTGTCCAACT-3′), RhoA (forward, 5′-CATCCGCTCCTTTGATGATCTT-3′; reverse, 5′-TGCTCGGGTCATGTTCAAGT-3′), GEF-H1 (forward, 5′- AGCCTGTGGAAAGACATGCTT-3′; reverse, 5′-TCAAACACTGTGGGCACATAC-3′) and GAPDH (forward, 5′-TCGGTGTGAACGGATTTG-3′; reverse, 5′-GGTCTCGCTCCTGGAAGA-3′). The transcript levels of the genes of interest were measured by qRT-PCR using the SYBR Green PCR mix (Applied Biosystems) in an Applied Biosystems 7300 detection system (Bio-Rad). The data were normalized to the expression levels of the cellular housekeeping gene GAPDH.

### RhoA activity assay

RhoA activity was measured by using a RhoA G-LISA absorbance-based biochemical assay kit (Cytoskeleton) according to the manufacturer's instructions. In brief, cells were lysed, aliquoted and snap frozen in liquid nitrogen. After rapid thawing, binding buffer was added to the cell lysate, which was subsequently incubated on a RhoA-GTP affinity plate coated with RhoA-GTP-binding protein in each well. The plate was placed on an orbital plate shaker at 400 rpm for 30 min at 4°C. After washes, primary anti-RhoA antibodies (1:250) and secondary HRP-linked antibodies (1:62.5) were sequentially added to the wells followed by an incubation on an orbital shaker at 400 rpm for 45 min at room temperature. Thereafter, the signal was developed with HRP-detection reagents. The absorbance was measured by means of a plate reader spectrophotometer Enspire (PerkinElmer). In the quantifications, the absorbance values of control cells were set to 1 in each experiment, and the differences between the control and the knockout, knockdown or transfection cells in corresponding experiments were calculated. The statistical differences between the two groups were assessed using the paired *t*-test. *P*<0.05 was considered significant.

### GEF activity assay

The activity of GEF-H1 was assayed by using GST–RhoA-G17A nucleotide-free mutants as described previously (Garcia-Mata et al., 2006). Cells were lysed in lysis buffer (150 mM NaCl, 20 mM HEPES, pH 7.4, 5 mM MgCl_2_, 1% Triton X-100, 1 mM DTT, 1 mM PMSF, 10 μg/ml aprotinin and leupeptin), incubated with 50 µg/ml glutathione–Sepharose-bound GST-RhoA G17A for 60 min at 4°C, and washed in the lysis buffer. Samples were subsequently analyzed by western blotting with the GEF-H1 antibody.

### Co-immunoprecipitation

Cells were harvested and lysed in NP-40 lysis buffer (50 mM Tris-HCl pH 7.4, 50 mM NaCl, 0.1% Triton X-100, 1% NP-40) plus 1× Roche complete protease inhibitors (Roche). Total cell lysate was used for immunoprecipitation. Primary GEF-H1 antibody (10 μg) was incubated with 50 μl (1.5 mg) of Dynabeads (Life Technologies) for 2 h at 4°C while under rotating. Cell lysate was then mixed with Dynabeads–antibody complexes and incubated overnight at 4°C while under rotating. After three 20 min washes with NP-40 lysis buffer, the protein–antibody complexes were eluted from the beads in 20 µl NuPAGE LDS sample buffer. Samples were subsequently analyzed by western blotting with the vimentin antibody.

### Polyacrylamide substrate preparation

Polyacrylamide (PA) gels were constructed and coated with fibronectin as described previously (Damljanovic et al., 2005). Briefly, coverslips were treated with 3-aminoplopyltrimethoxy silane, dried and soaked in 0.5% glutaraldehyde. In order to prepare gels of 33 kPa stiffness, acrylamide (40%, Bio-Rad), Bis (2%, Bio-Rad), HEPES (1 M) and distilled water were mixed in concentrations of 5% acrylamide with 0.12% Bis. Gels were crosslinked using ammonium persulfate (10%) and TEMED. A 15 μl of the mixture was allowed to polymerize between the activated coverslip and a normal coverslip. PA gels needed for the experiments were constructed concurrently and used immediately. PA gels were activated for protein cross-linking with sulfo-SANPAH (Pierce Biotechnology) under a UV lamp and coated with fibronectin (10 µg/ml) at 4°C overnight. Prior to experiments, the gels were covered with PBS in UV lamp for 30 min to sterilize and then equilibrated with DMEM at 4°C, 5% CO_2_, for 45 min.

### Traction force microscopy

Both control and vimentin-knockout cells were cultured for 3–8 h on custom-made 35 mm dishes (Matrigen) with fibronectin-coated polyacrylamide gel (elastic modulus 25 kPa). 200 nm YG fluorescent (505/515) microspheres were immobilized to the surface of the gel as described previously (Marinkovic et al., 2012). Using an inverted fluorescence microscope (3I Marianas), images of the cells and of the fluorescent microspheres directly underneath the cells were acquired during the experiments and after cell detachment with trypsin. By comparing the reference image with the experimental image, we computed the cell-exerted displacement field. From the displacement fields, and manual traces of the cell contours, together with knowledge of substrate stiffness, we computed the traction force fields using the approach of constrained Fourier-transform traction cytometry (Butler et al., 2002). From the traction fields, we calculated the root mean squared values (RMS) of tractions. Because tractions vary log-normally (Krishnan et al., 2009), statistical differences in RMS traction between the control and vimentin-knockout groups were assessed by using the Mann–Whitney–Wilcoxon rank-sum (MWW) test. *P*<0.05 was considered significant.
